# Two novel homozygous mutations of *CAPN1* in Chinese patients with hereditary spastic paraplegia and literatures review

**DOI:** 10.1186/s13023-019-1053-1

**Published:** 2019-04-25

**Authors:** Fang Peng, Yi-Min Sun, Chao Quan, Jian Wang, Jian-Jun Wu

**Affiliations:** 1 Department & Institute of Neurology, Huashan Hospital, Fudan University, 12 Wulumuqi Zhong Road, Shanghai, 200040 China; 2 Department of Neurology, Jing’an District Center Hospital of Shanghai, 259 Xikang Road, Shanghai, 200040 China

**Keywords:** Hereditary spastic paraplegias (HSP), Spastic paraplegia 76(SPG76), *CAPN1* mutations, Ataxia

## Abstract

**Background:**

Hereditary spastic paraplegias (HSP) are of great clinical and genetic heterogeneity. According to the clinical features, HSP can be divided into pure or complicated subtypes which combined with other neurological symptoms including cerebellar ataxia. Up to date, 78 loci or genes have been implicated in HSP. *CAPN1* was a novel gene detected recently for spastic paraplegia 76 (SPG76).

**Methods:**

Patients referred to our clinic with spastic or spastic-ataxic gait were collected. Genetic testing of the probands were performed by target sequencing of a panel containing over 4000 known virulence genes. And the candidate mutations were further confirmed by polymerase chain reaction (PCR) and Sanger sequencing. The clinical materials of these patients were demonstrated retrospectively.

**Results:**

Two Chinese patients, both from consanguineous families, each carried a novel homozygous mutation of *CAPN1*, p.R48X and p.R339X. The male proband presented pure HSP subtype while the female proband presented complicated HSP symptoms with cerebellar ataxia. We then reviewed all the literatures of HSP patients carrying *CAPN1* mutations and summarized the molecular spectrum and clinical characteristics of *CAPN1*-related SPG76.

**Conclusion:**

These two SPG76 patients carrying *CAPN1* mutations were the first reported in China. By reviewing the clinical manifestations of SPG76 patients, we validated the “spastic-ataxia” phenotype and emphasized the association between spasticity and ataxia, indicating the importance of *CAPN1* screening in HSP patients.

## Introduction

Hereditary spastic paraplegias (HSP) present great genetic and clinical heterogeneity, mainly manifesting as spasticity and weakness in the lower limbs [1]. On the basis of clinical features, HSP can be categorized into pure and complicated subtypes [2]. In addition to the dominant progressive spasticity and weakness, pure HSP can also present symptoms of hypertonic bladder and sensory disturbances. Complicated HSP is often accompanied by other neurological symptoms, including cerebellar ataxia, seizure, extrapyramidal signs, intellectual disability, peripheral neuropathy, amyotrophy, optic atrophy and others [3, 4]. Among them, cerebellar ataxia occurs most frequently in complicated HSP, resulting in “spasticity-ataxia” phenotype [5]. The hereditary modes of HSP include autosomal-dominant (AD), autosomal-recessive (AR), X-linked and maternal trait of inheritance which due to mitochondrial impairment [4]. In all these hereditary modes, AR inheritance is the commonest one [6]. Up to date, a total of 78 loci have been implicated in HSP [5].

Recently, *CAPN1* has been identified as a causative gene for spastic paraplegia 76 (SPG76, MIM#616907, NM_005186), a complicated form of HSP [7]. The protein encoded by *CAPN1* was calpain-1, which was widely expressed in central nervous system (CNS), has been involved in several important functions of synaptic plasticity, synaptic restructuring, axon maturation and maintenance [8–10]. In 2016, mutations of *CAPN1* [c.884G > C (p.R295P), c.1579C > T (p.Q527*), c.406delC (p.P136Rfs*40) and c.1605 + 5G > A] were identified in three AR inherited HSP pedigrees for the first time [7]. Subsequently, other homozygous or compound-heterozygous mutations of *CAPN1* were reported in other groups [11–17].

**Fig. 1:**
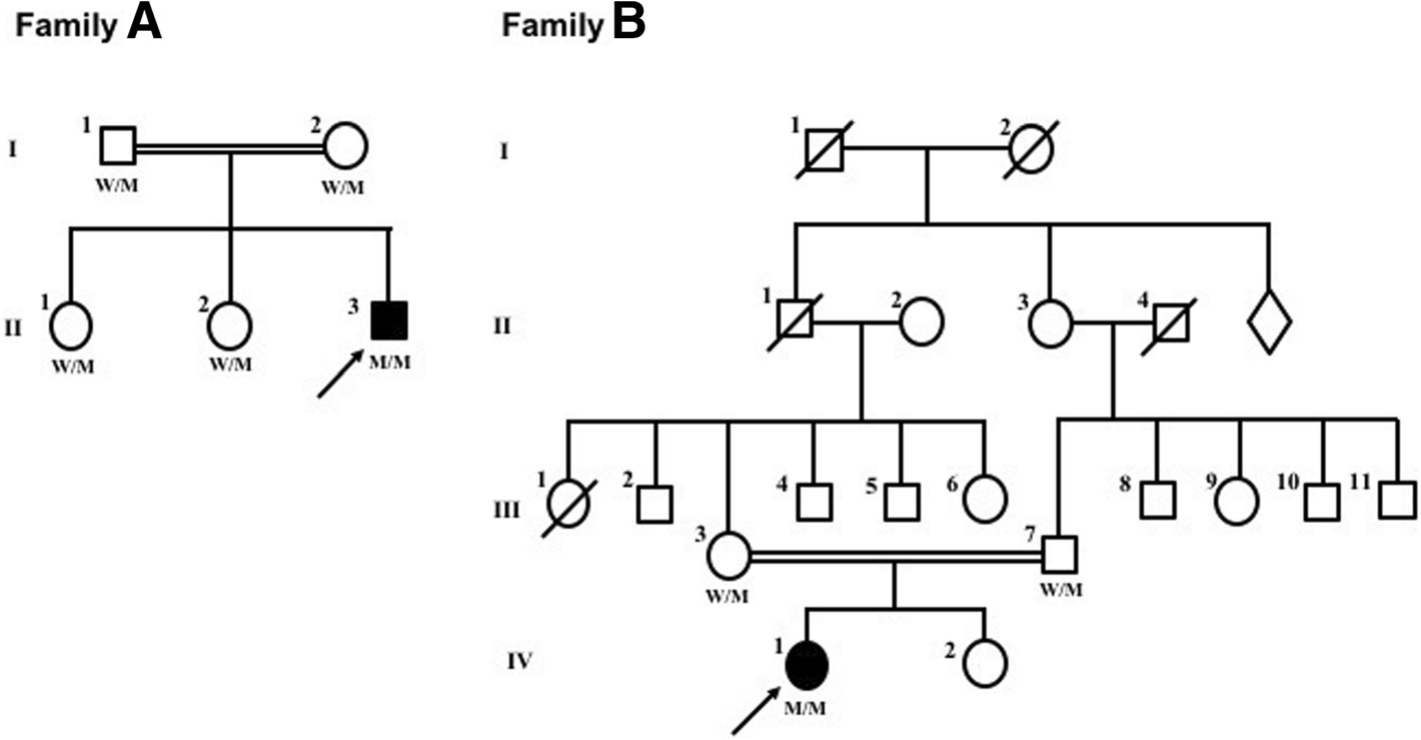
Pedigrees of family A and family B with *CAPN1* mutations. Arrow: proband; square: male; circle: female; slash: deceased; solid symbol: affected. W/M and M/M represent the genotype of *CAPN1*. W/M: wild type/mutant; M/M: mutant/mutant

In this study, we reported two Chinese HSP probands, both from consanguineous family (Fig. 1), each carried a novel homozygous mutation of *CAPN1.* To our knowledge, they were the first SPG76 patients reported in China. Their clinical features and disease progressions were demonstrated retrospectively and would broaden the molecular and clinical spectrum of Chinese HSP patients.

## Methods

The probands with walking problems such as spastic or spastic-ataxic gait were collected in the Neurogenetics clinic in Huashan Hospital (Shanghai, PRC). The clinical materials were investigated in both probands.

Genomic DNA was extracted from peripheral blood of both patients and their parents or siblings. Genetic testing of the probands were performed by target sequencing of a panel containing over 4000 known virulence genes. The sequencing was carried out by Illumina HiSeq X-ten platform. The variants screen protocol was as previously reported [18]. The candidate mutations were further confirmed by polymerase chain reaction (PCR) and Sanger sequencing. These mutations were also performed in the parents or siblings to confirm the family co-segregation.

Written informed consents were obtained from both patients and their relatives. This study was approved by the ethics committee of Huashan Hospital.

## Results

### Results of genetic testing

In family A, the mean depth of target sequencing was 73.5X and the coverage was 100%. The percentage of the target region with mean depth > 20X was 97.0%. According to the screening criteria of low variants frequencies [< 1% in 1000Genome (http://www.1000genomes.org/home), ExAC (http://exac.broadinstitute.org/)] and homozygous mapping, 38 variants were left. But after further screened by clinical manifestations, only one novel homozygous mutation of c.142C > T (p.R48X, NC_000011.10:g.64950314C > T) in *CAPN1* (NM_001198868) was found with the depth of 84X.

In family B, the mean depth of target sequencing, the coverage and the percentage of the target region with mean depth > 20X was 107.8X, 99.3 and 97.4% respectively. After screened by the criteria mentioned above, one novel homozygous mutation of c.1015C > T (p.R339X, NC_000011.10:g.64956067 C > T) in *CAPN1* was found with the depth of 70X.

Both mutations have been confirmed in the probands and their parents or siblings by Sanger sequencing. The unaffected parents and two unaffected elder sisters of the proband in Family A all carried c.142C > T in the heterozygous state. In family B, the mutation of c.1015C > T was found heterozygous in the unaffected parents of the proband.

**Fig. 2:**
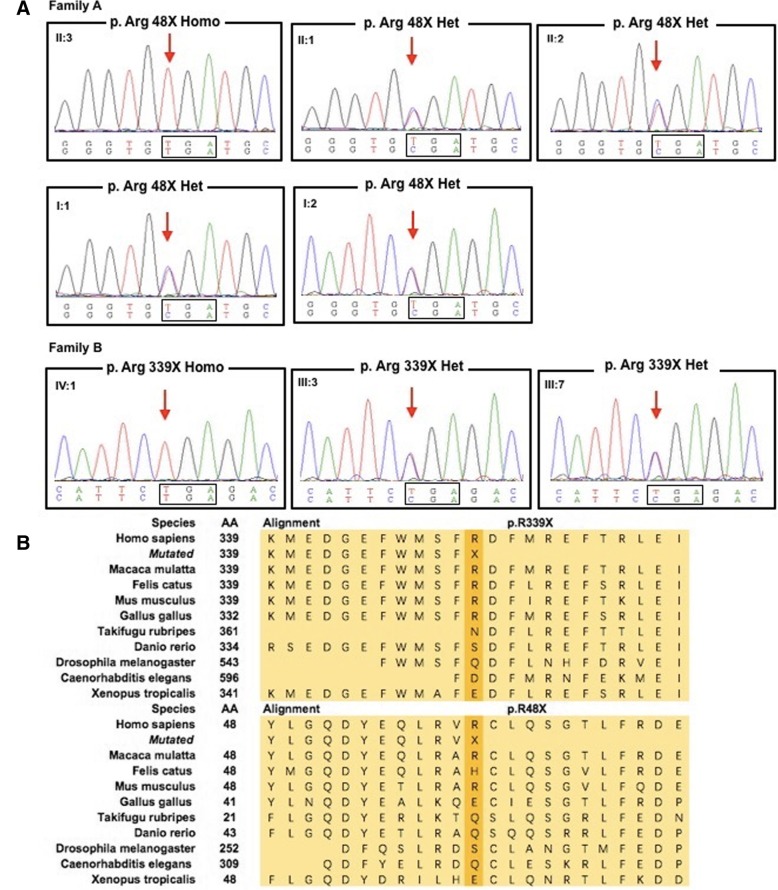
**a** Sanger sequencing of both probands and their other unaffected family members. **b** Sequence alignment of calpain-1 across species

Both variants were partly conserved across species (Fig. 2) and was predicted to be disease causing by mutationtaster (mutationtaster.org) since the truncated mutant took place in positions of R48 and R339 which might cause nonfunctional protein product or affect functional subdomains of the protein.

### Clinical characteristics

The proband from family A gradually developed walking difficulties and stiffness in the lower limbs at age 18. The symptoms deteriorated slowly. He came to our clinic at age 38 and shown a typical spastic gait. Neurologic examination revealed that the muscle tone in both lower limbs was extremely high. Knee and ankle hyperreflexia were also found in both lower limbs. Bilateral Hoffmann sign and Babinski sign were positive. He could still walk along a straight line. Finger-to-nose test and diadochokinesia were performed well. The examination of ocular movement was fine. No distal sensory impairment, cognitive deterioration, bladder dysfunction, or dysarthria was complained. Nerve conduction study and electromyography did not reveal any neurogenic and myogenic damages. The results of head and spinal cord magnetic resonance image (MRI) were also negative.

The proband from family B referred to our clinic for progressive walking difficulties at age 41. Five years ago, she reported weakness in the lower limbs and there was a slight tiptoe when she was walking. She felt slight imbalance and could not walk along a straight line well. The neurological examination revealed hyperreflexia in four limbs and positive bilateral Hoffmann sign. She presented with a moderately spastic-ataxic gait. She had slight bilateral dysmetria when performed finger-to-finger test and mild dysdiadochokinesia. The heel knee test was fine. The ocular pursuit and saccades were normal. She scored 6/40 on the Scale for the Assessment and Rating of Ataxia (SARA). The mini-mental state examination score was 29/30 (education year of 14) suggesting the normal cognitive function. No dysarthria, distal sensory impairment or bladder dysfunction was reported. Neither cerebellum nor spinal cord showed significant atrophy on MRI.

### Literatures review

A total of nineteen pedigrees including 35 patients (24 Female, 11 Male patients) with *CAPN1* mutations reported till recently were reviewed (Table 1). All the patients showed a pattern of AR inheritance and 85.7% pedigrees were consanguineous. Thirty patients carried homozygous mutations and five patients carried compound heterozygous mutations. The onset age ranged from five to 39 years old. With all the available clinical materials, lower limbs spasticity, presenting with stiffness, hyperreflexia and pathological signs, developed in around 80% patients, followed by cerebellar ataxia developing in 62.9% of the cases, dysarthria in 51.4%, skeletal or tendon deformity in 31.4%. Weakness in lower limbs and ocular movement disorder could also be seen. Some patients developed abnormal bladder function, dysphagia, peripheral neuropathy, intention tremor and even other uncommon symptoms.

**Table 1:** Literatures review of reported HSP patients with *CAPN1* mutations

Study (year)	Case No.	Gender	Population	Consanguinity	Mutations	Het/Hom	Exon	Transcript	Age at onset (year)	Clinical features											MR or CT imaging (Brain or spine)
										Lower limbs spasticity	Lower limbs hyperreflexia	Upper limbs hyperreflexia	Babinski sign	Skeletal or tendon deformity	Weakness or amyptrophy	Ocular movement disorder	Abnormal bladder function	Dysarthria	Ataxia	Additional symptoms	
Gan-Or Z, et al. (2016) [7]	3	M	Mornoccan	Y	C.884G > C (p.R295P)	Hom	exon8	NM_005186	NA	NA	NA	NA	NA	NA	NA	NA	NA	NA	NA	NA	NA
		F							20	+	+	+	+	+	+	–	+	+	–	NA	NA
		F							NA	NA	NA	NA	NA	NA	NA	NA	NA	NA	NA	NA	NA
	5	M	Mornoccan	Y	C.1579C > T (p.Q527*)	Hom	exon14		35	+	+	+	+	–	+	–	–	+	–	hypoesthesia, peripheral, neuropathy, dysarthria, akinetic face, abolished sympathetic skin reflex in lower limbs	NA
		F							36	+	+	+	+	+	+	–	–	+	+	peripheral neuropathy, facial hypokinesia, abolished sympathetic skin reflex in lower limbs	NA
		M							22	+	+	+	+	+	+	–	–	+	–	NA	–
		M							39	+	+	+	–	–	–	+	–	+	+	NA	NA
		F							24	+	+	+	+	+	–	–	–	+	–	abolished sympathetic skin reflex in lower limbs	NA
	2	M	Ladho and Utah	N	C.406delC (p.P136Rfs*40) c.1605 + 5 G > A	Com-het	exon4 exon14		33	+	NA	NA	+	+	NA	–	–	NA	–	NA	mild atrophy of cervical spinal cord
		F							19	+	+	+	+	+	+	–	+	NA	+	NA	slightly prominent sulci
Wang Y, et al. (2016) [11]	2	F	Bangladeshi	Y	c.337 + 1 G > A	Hom	exon3	NM_001198868	Late teens	+	+	NA	NA	NA	NA	NA	NA	+	+	dysphagia, mild cognitive decline	mild cerebellar atrophy
		F							NA	NA	NA	NA	NA	NA	NA		NA	NA	NA	NA	NA
	1	F	Italian	NC	c.183dupC (p.F61 fs)	Hom	exon2		25	+	+	+	+	NA	NA	NA	+	+	+	dysphagia, bilateral positive Hoffmann’s reflex	–
	2	F	Tunisian	Y	c.1534C > T (p.R512C)	Hom	exon13		23	+	+	+	NA	–	NA	NA	NA	+	+	NA	cerebellar atrophy
		F							20	+	+	+	NA	–	NA	NA	NA	+	+	NA	NA
	1	F	French	Y	c.463C > T (p.Q155X) c.1142C > T (p.A381V)	Com-het	exon5 exon10		20	+	+	+	+	+	+	+	+	+	+	dysphagia, hypokinesia, vibration sense at ankles decreased, bilateral positive Hoffmann’s reflex	cerebellar atrophy, white matter changes, mild vermian atrophy
Travaglini,L, et al. (2017) [12]	1	M	Italian	N	c.221G > A (p.G74D) c.911C > T (p.T304 M) c.1418G > T (p.R473L)	Com-het	exon2 exon8 exon13	NM_001198868	5	+	+	NA	+	NA	NA	NA	NA	NA	NA	spastic hypertonia	–
Tadic V, et al. (2017) [13]	2	F	NA	Y	c.759 + 1 G > A	Hom	exon6	NM_001198868	29	+	+	NA	+	+	NA	+	NA	+	+	muscle hypertonic	cerebellar vermal atrophy
		F							33	NA	+	NA	±	+	NA	NA	NA	NA	+	slight intention tremor	NA
Kocoglu C, et al. (2018) [15]	1	F	NA	NA	c.994G > A (p.G332R)	Hom	exon9	NM_001198868	21	+	NA	NA	NA	+	+	NA	NA	+	+	upper limb spasticity keratoconus	–
	2	F	NA	Y	c.1176G > A (P.R392*)	Hom	exon10		15	+	+	NA	+	+	NA	NA	NA	+	+	upper limb spasticity	–
		F							15	+	+	NA	+	NA	NA	NA	NA	+	+	NA	NA
Lambe J, et al. (2018) [14]	1	F	Irish	N	c.1534C > T (p.R512C)	Hom	exon13	NM_001198868	14	+	+	+	+	NA	NA	–	NA	–	+	NA	midbrain pons, cerebellar atrophy, spinal cord normal
Shetty A, et al. (2018) [16]	1	F	Japanese	Y	c.2118 + 1G > T	Hom	exon21	NA	37	NA	NA	NA	NA	NA	NA	NA	NA	NA	+	upper motor neuron findings in the legs	NA
	2	M	Turkish	Y	c.397C > T	Hom	exon4	NA	23	NA	NA	NA	NA	NA	NA	NA	NA	NA	NA	progressive spastic paraparesis	NA
		F						NA	20	NA	NA	NA	NA	NA	NA	NA	NA	NA	NA	severe proximal weakness	NA
	1	M	Punjabi	Y	c.843 + 1G > C	Hom	exon7	NA	37	+	NA	NA	NA	NA	NA	NA	NA	NA	+	spastic quadriparesis	NA
Melo US. et al. (2018) [17]	3	F	Brazilian	Y	c.1176G > A (P.R392*)	Hom	exon10	NM_001198868	NA	+	NA	NA	NA	NA	NA	NA	NA	+	two of three	NA	NA
		F							NA	+	NA	NA	NA	NA	NA	NA	NA	+		NA	NA
		M							NA	+	NA	NA	NA	NA	NA	NA	NA	+		NA	NA
	1	F		Y	c.1176G > A (P.R392*)	Hom	exon10		22	+	+	NA	NA	NA	NA	NA	NA	NA	**–**	NA	NA
	2	F		Y	c,675C > A p.Y225*	Hom	eoxn6		20	+	NA	NA	NA	NA	NA	NA	NA	NA	+	NA	NA
		M							35	+	NA	NA	NA	NA	NA	NA	NA	NA	+	NA	NA
	2	F		N	c.1176G > A (P.R392*) c.618_619 delAG (p.G208 Qfs*7)	Com-het	exon10 exon6		30	+	NA	NA	NA	NA	NA	NA	NA	NA	+	NA	NA
		M		Y	c.1176G > A (p.R392*)	Hom	exon10		38	+	NA	NA	NA	NA	NA	NA	NA	NA	+	NA	NA
Current study	2	M	Chinese	Y	c.142C > T (p.R48*)	Hom	exon2	NM_001198868	18	+	+	+	+	–	–	–	NA	–	–	muscle hypertonic in lower limbs, bilateral positive Hoffmann’s reflex	–
		F		Y	c.1015C > T (p.R339X)	Hom	exon10		41	+	+	+	–	–	+	–	–	–	+	bilateral positive Hoffmann’s reflex	–

*AR* autosomal-recessive, *com-het* compound heterozygous, *CT* computed tomography, *hom* homozygous, *HSP* Hereditary Spastic Paraplegia, *F* female, *M* male, *MRI* magnetic resonance image, *N* no, *NA* not available, *NC* not certain, *Y* yes, *y* years old, +: positive, −: negative or normal, ±:suspicious

## Discussion

With the wildly application of next generation sequencing, more and more classical “HSP genes” causing cerebellar ataxia were found and vice versa. So, these genes could be categorized as “spasticity-ataxia” spectrum. According to a review in 2017, genes related to “spasticity-ataxia” spectrum was expanded to 69 members [5]. *CAPN1* was one of them, manifesting as pure HSP or complicated HSP. The mutations in *CAPN1* causing autosomal recessive HSP have been found since 2016 by whole exome sequencing in three pedigrees. Among these patients carrying *CAPN1* mutations, lower limbs spasticity was the predominant symptom combined with cerebellar ataxia or not. Therefore, “spasticity-ataxia” phenotype might conduce to the diagnosis of SPG76.

The protein product of *CAPN1*, calpain-1, also known as μ-calpain, contains four domains: the N-terminal anchor helix region, the CysPc protease domain (including two protease core domains of PC1 and PC2), the C2 domain-like domain and the penta-EF-hand domain (PEF). As a calcium-activated cysteine protease, calpain-1 binds to calcium through PEF domain [19]. It has been proved that the activation of calpain-1 is required for its neuro-protective role in CNS [20]. Several mechanisms for the protective role were suggested by interacting with CDK5 and NR2B to control NMDA-receptor degradation [21] or affecting AMPA receptors through degradation of its substrate, glutamate receptor-interacting protein [22]. In calpain-1 deficient mice, dysfunction of calpain-1 reduced dendritic branching complexity and led to spine density deficits [23]. In zebrafish embryos, knockdown of calpain-1 induced disruption of microtubule network in brain and spinal cord [24], which indicated that dysfunction of calpain-1 could result in neurodegeneration or disorganization of neurons [25]. Immunohistochemistry study revealed that calpain-1 was the major calpain isoform in cerebellar neurons [26], and the activity of it in cerebellum was higher than that in cortex or hippocampus [27], suggesting that calpain-1 played a key role in maintaining the normal cerebellar function.

All the reported mutations scattered in exons 2–6, 8–10, 13, 14 and 21of *CAPN1*, potentially damaged the normal structure of calpain-1 or led to early termination of protein coding, causing the dysfunction of calpain-1. In this current study, the probands were in accordance with two HSP subtypes: the male patient presented with pure HSP subtype with normal cerebellar function, while the female patient manifested as classical complicated HSP subtype showing symptoms of both HSP and cerebellar ataxia. Two novel homozygous mutations c.1176G > A and c.675C > A of *CAPN1* were detected respectively. These two mutations were situated in exon 2 and exon 10 and brought on a premature stop codon at the positions of R48 and R339, causing the destruction of calpain-1 normal structure. The structural incompleteness of calpain-1 would interfere with its neuro-protective role in CNS and induce neurodegeneration or disorganization of neurons, which might lead to SPG76.

## Conclusion

Together with previously reported cases, our study broadened the clinical and molecular spectrum of *CAPN1*-related SPG76 and exemplified the concept of “spasticity-ataxia” phenotype, further increasing our understanding of complicated HSP form and its connection with cerebellar ataxia. All these observations indicated that *CAPN1* screening is necessary in HSP patients, especially when patients suffer from spasticity-ataxia phenotype.

## Abbreviations

AD: Autosomal-dominant
AR: Autosomal-recessive
CNS: Central nervous system
HSP: Hereditary spastic paraplegias
MRI: Magnetic resonance image
PCR: Polymerase chain reaction
PEF: Penta-EF-hand domain
SARA: Scale for the Assessment and Rating of Ataxia
SPG76: Spastic paraplegia 76
